# Identifying Value Factors in Institutional Leaders’ Perspectives on Investing in Health Professions Educators

**DOI:** 10.1001/jamanetworkopen.2022.56193

**Published:** 2023-02-16

**Authors:** Ann Poncelet, Sally Collins, Darren Fiore, Glenn Rosenbluth, Helen Loeser, George F. Sawaya, Arianne Teherani, Anna Chang

**Affiliations:** 1 University of California, San Francisco, San Francisco, California; 2 University of California, San Francisco, School of Medicine, San Francisco, California

## Abstract

**Importance:**

Investing in educators, educational innovation, and scholarship is essential for excellence in health professions education and health care. Funds for education innovations and educator development remain at significant risk because they virtually never generate offsetting revenue. A broader shared framework is needed to determine the value of such investments.

**Objective:**

To explore the value factors using the value measurement methodology domains (individual, financial, operational, social or societal, strategic or political) that health professions leaders placed on educator investment programs, including intramural grants and endowed chairs.

**Design, Setting, and Participants:**

This qualitative study used semi-structured interviews with participants from an urban academic health professions institution and its affiliated systems that were conducted between June and September 2019 and were audio recorded and transcribed. Thematic analysis was used to identify themes with a constructivist orientation. Participants included 31 leaders at multiple levels of the organization (eg, deans, department chairs, and health system leaders) and with a range of experience. Individuals who did not respond initially were followed up with until a sufficient representation of leader roles was achieved.

**Main Outcomes and Measures:**

Outcomes include value factors defined by the leaders for educator investment programs across the 5 value measurement methodology domains: individual, financial, operational, social or societal, and strategic or political.

**Results:**

This study included 29 leaders (5 [17%] campus or university leaders; 3 [10%] health systems leaders; 6 [21%] health professions school leaders; 15 [52%] department leaders). They identified value factors across the 5 value measurement methods domains. Individual factors emphasized the impact on faculty career, stature, and personal and professional development. Financial factors included tangible support, the ability to attract additional resources, and the importance of these investments as a monetary input rather than output. Operational factors identified educational programs and faculty recruitment or retention. Social and societal factors showcased scholarship and dissemination benefits to the external community beyond the organization and to the internal community of faculty, learners, and patients. Strategic and political factors highlighted impact on culture and symbolism, innovation, and organizational success.

**Conclusions and Relevance:**

These findings suggest that health sciences and health system leaders find value in funding educator investment programs in multiple domains beyond direct financial return on investment. These value factors can inform program design and evaluation, effective feedback to leaders, and advocacy for future investments. This approach can be used by other institutions to identify context-specific value factors.

## Key Points

**Question:**

What are the value factors that university and health systems leaders deem important regarding intramural programs that invest in health professions educators?

**Findings:**

In this qualitative study of 29 leaders at a university and affiliated health systems, thematic analysis revealed value factors for educator investment programs using the value measurement methods framework (individual, financial, operational, political or strategic, and social or societal). Participants described value in multiple domains beyond direct financial return on investment.

**Meaning:**

These findings suggest that value factors can inform program design and evaluation, feedback to leaders, and advocacy for future investments, and can be used by other institutions to identify context-specific value factors.

## Teaser

This qualitative study explores the value factors that health professional leaders placed on educator investment programs, including intramural grants and endowed chairs, through interviews at an urban academic health professions institute in the US.

## Introduction

Investing in educators, educational innovation, and scholarship is essential for excellence in medical education, a core mission of all medical schools and health care.^1,2^ Studies demonstrate the benefit of intramural grants and endowed chair programs to outcomes like education innovation and educator growth.^3,4^ Yet, funding for medical education, including for innovation and the educators themselves, remains at significant risk as academic medical centers (AMCs) face declining revenue from the clinical and research enterprises along with increasing costs of undergraduate and graduate medical education.^2,5^ Because tuition rarely covers the costs of medical education,^2^ we must engage institutional leaders to advocate for support.^6^

Funds for educator investment programs (EIPs) like intramural grants or endowed chairs are often allocated by leaders with backgrounds in clinical administration or biomedical research. They hold an understandable economic focus since clinical revenue currently subsidizes the AMC’s education and research missions.^2,7^ However, economic metrics, such as return on investment (ROI), are insufficient to determine the value of EIPs.^7^ AMC leaders and educators need a broader framework to inform future dialogue and resource allocation. To date, the perspective of institutional leaders is missing, especially regarding the factors that have value beyond financial ROI.

One framework for exploring value factors beyond traditional financial metrics is the value measurement methodology (VMM) developed by the Federal Chief Information Council.^8^ Used by the US federal government, VMM presents value factors in 5 domains: individual (benefits individual recipient), financial (including costs, revenues), operational (improves system operations), social or societal (benefits group or society), and strategic or political (advances institution’s mission, strategic goals, priorities, and mandates).^8^ One clinical simulation program used VMM to identify value factors in additional domains, such as operational (decreased length of stay) and strategic (patient safety culture).^9^ This framework has potential and has yet to be applied to academic EIPs. Using the VMM framework, we present an exploration of value factors of leaders at a health professions institution and affiliated health systems regarding intramural programs that invest in educator growth and education innovation.

## Methods

This qualitative study used thematic analysis^10,11^ to identify themes in interview data, with a constructivist orientation, to elucidate the perspectives of institutional leaders. The institutional review board at the University of California, San Francisco, deemed this study exempt via the Common Rule. Informed consent was obtained orally and via email. This study followed the Standards for Reporting Qualitative Research (SRQR) reporting guideline.

### Setting and Participants

Our study was conducted at the University of California, San Francisco (UCSF), an academic health professions institution with 4 schools—dentistry, medicine, nursing, and pharmacy—and a physical therapy department. We invited 55 leaders via email from across UCSF and 3 affiliated health systems. We chose leaders at multiple levels of leadership with a range of experience in their roles, with UCSF and the EIP programs, and from departments of different specialties and sizes to reach a sufficient depth of understanding.^12^ We followed up with those who did not initially respond until we had sufficient representation of leader roles at 31 respondents, 2 of whom served as pilot interviewees.

### Instrument

We developed a semistructured interview guide (eAppendix in Supplement 1) based on 5 VMM domains (financial, individual, operational, social or societal, and strategic or political),^8^ adapted for education. We highlighted as exemplars 2 EIPs developed by the UCSF Haile T. Debas Academy of Medical Educators (AME)^3,4,13^: the Innovations Funding for Education program (IF), and the Endowed Chair program (EC) at $15 000 to $30 000 per year. The IF supports UCSF health professions educators to develop, pilot, and study curricular innovations with competitive intramural grants.^3^ The EC supports the career development of AME members, with the aim of expanding their impact within and beyond the institution.^4^ We gave each leader a list of IF recipients and EC holders from their department or unit to illustrate these programs and serve as a prompt. We also asked leaders to articulate the most important factor.

Two senior leaders served as pilot interviewees to refine the interview guide, train 3 author-interviewers (A.C., D.F., G.R.), and ensure consistency. These data are not included in the analysis. The remaining 29 interviews were conducted between June and September 2019; no one interviewed a leader to whom they reported directly. Interviews lasted 12 to 59 minutes (mean [SD], 35.7 [8.8] minutes) and were conducted in-person using a mobile phone voice recording application or virtually using Zoom videoconferencing. All interview audio recordings were transcribed.

### Reflexivity

This study used a set of continuous, collaborative, and multifaceted practices through which we self-consciously critique, appraise, and evaluate how our subjectivity and context influence the research processes. Six authors are clinician educators (A.P., A.C., G.R., D.F., G.S., H.L.), 1 is a is an education researcher (A.T.), and 1 is an education research associate (S.C.). Five authors held endowed chairs prior to or at the time of the study (A.C., D.F., G.R., G.S., A.P.), and 3 (A.C., G.R., A.T.) had previously directed or codirected the IF program. One author is the current director of the AME (A.P.), and another a former director (H.L.). Three authors have never been a recipient of these programs (S.C., A.T., H.L.). We engaged in regular, reflective discussion, which enabled us to consider or challenge each other’s assumptions when interpreting findings.

### Data Analysis

We used thematic analysis,^10,11^ following an inductive approach, to analyze data once all interviews had been transcribed. Four authors (A.P., S.C., A.T., G.S.) developed a codebook from preliminary, low-inference codes (observable data expressed in a nonjudgmental way) based on the interview questions, which was further refined with codes generated from the data through an iterative consensus-building approach using 3 interview transcripts. These 4 authors then coded the rest of the transcripts. Each transcript was coded and reconciled by 2 of these team members through discussion to achieve consensus. Five authors (A.C., A.P., G.R., D.F., S.C.) reviewed the analyzed data to synthesize overarching themes. We drew on the perspectives of all team members throughout the analysis to facilitate a shared understanding of the data and ensure that the themes represented the voices of the leaders, not the perspectives of the study team. We organized coded transcripts with Dedoose analytic software version 9.0.62 (SocioCultural Research Consultants).

## Results

We interviewed 29 leaders for analysis (5 [17%] campus or university leaders; 3 [10%] health systems leaders; 6 [21%] health professions school leaders; 15 [52%] department leaders) (Table 1). We present the themes that emerged using VMM framework domains: individual, financial, operational, social or societal, and strategic or political (Table 2). We begin with the impact on the individual beneficiary of EIPs and conclude with the value factors leaders identified as the most important.

**Table 1.** Characteristics of Institutional Leader Participants^a^

Level	Senior leader role (all full professors)	Years in role		Total
		≤5 y	>5 y	
Campus/university	Leaders have authority across all 5 schools plus the UCSF health system	3	2	5
Health system	Leaders have authority over their specific affiliate site and/or health system	2	1	3
Health professions school	Leaders have authority across their specific school	4	2	6
Department	Leaders have authority over their specific department	12	3	15
Clinical departments				11
Basic science departments				4

Abbreviation: UCSF, University of California, San Francisco.

^a^ Characteristics of 29 leader participants whose data was included in the analysis.

**Table 2.** Value Factors, Themes, and Quotes From Institutional Leaders on Investing in Health Professions Educators

Value factor: themes	Representative quotes from institutional leaders
**Individual**	
Career/stature	The value is in what it did for the faculty member in promoting their career…also part of the goal is to position these folks in a way that makes them more competitive for leadership roles, if that’s what they aspire to. (Participant 15-department level)
	The impact of individuals and their ability to be successful, and the ability for them to identify what it is that they really want to focus their career on, is, to me, probably, the most important piece. I just feel so strongly that if you can support people to identify what they’re passionate about, and that fits in their space;…that there is no way anyone’s going to lose in that path, right? The learners are going to win, the individual faculty members are going to win, the department’s going to win because it’s going to continue to be successful; it’s good for the institution. (Participant 3-department level)
Development	I think the most important is the development of the individual educator. The chair-holder, the project lead, the idea that their interests could be further developed, that there’s a whole world out there of education that they can get involved in and that then another, they can have that. They can convey that opportunity to other faculty members. (Participant 20-department level)
**Financial**	
Actual Funds/time/responsibility	So, for us, a lot of our young faculty who want a career as a clinician educator, this is the only way they can get projects off the ground. (Participant 23-department level)
Money as input	I think that ...dollars and cents are not the best way to measure value. I mean they’re critical, but, if you look at a corporation, they measure value by dollars because they’re supposed to return money to their shareholders….But what we need to do is, our value is, are we enhancing the dissemination of our mission to the larger community? And so, money is crucial. Everything costs money, but money is an input, not an output. (Participant 1-campus/university level)
**Operational**	
Educational programs	All the different things that the academy chairs have done, and then all of the different small grants that have been funded. It’s probably hard to do a true return on investment calculation on that kind of thing. But I think as you look at the totality over a period of almost 2 decades, it has to add up to better teachers, a better student experience, and hopefully, better course content. (Participant 13-campus/university level)
	For accreditation standards….one particular value that I like is when they do the end-of-year summary, and they collect the various educational endeavors that people have taken part of, and they aggregate it into a report. (Participant 4-department level)
Efficiency	I’m constantly looking at the cost of education and the debt that students acquire for an education....But....how can we educate someone and do it even more efficiently and effectively so that we decrease the cost for them. (Participant 10-health professions school level)
Recruitment/retention	I see it as a great opportunity for us to retain people that otherwise we might not be able to retain, to recruit people that otherwise we might not be able to recruit, all to the betterment of our system, all to the betterment of the trainee educational environment. So those are the 2 things that come to mind that are top of mind for me, recruitment and retention. (Participant 21-health system level)
**Social and societal**	
Dissemination	I think the second thing would be to track the connection between these types of programs with substantial changes in the way medical education beyond our organization is conducted. (Participant 26-health professions school level)
External community	I think that programs like this are part of our compact, or our contract with society, which says that we’re going to do everything humanly possible to find the best talent and give them the resources and time, and opportunity to ask these questions, and train the next generation so that there’s another group of people who can carry this onwards. (Participant 12-campus/university level)
	….the more that we can advance the quality of health education, the importance of it to the population, so not just internally but within the community that our organization serves. The more that this can demonstrate the value of it, and then expand it to the community, the better. So, to me, the value is more of a societal value. (Participant 8-campus/university level)
Internal community	For a faculty member who’s doing a project or for a person who’s holding a chair to be part of a community of medical educators that are considering the broad array of challenges in medical education is very valuable….the projects that are going on in our department and others that have a halo effect, they are best practice, they are new ideas that get developed. And it’s kind of an incubator for that that filters back to the department. (Participant 20-department level)
	And then, I think again, sort of harnessing this, the inter-professional, inter-educational connection strategically I think can be very important. (Participant 17-department level)
**Strategic and political**	
Culture and symbolism	[These] programs....might not be the traditional place to sink money, but there is a quite tangible cultural, strategic, psychological benefit and an affirmation that goes with planting that flag. (Participant 5-department level)
	Education is unique in that it essentially does not generate any new income….And that’s why I think the identification and commitment of resources in this way...is not only a real indication of support for the education mission….it’s also symbolic. It also shows that we really do consider this as equal to the other missions. (Participant 12-campus/university level)
Innovation	If you can use a small amount of money to spark someone to do something creative that may be generalizable, either across the university or across other institutions, then you’re really making a fundamental change. (Participant 10-health professions school level)
	Getting a grant is a very clear indication of your capacity for creativity. And creativity is the currency of academia. (Participant 12-campus/university level)
Organizational success	We can look at this university and say, “Well, it’s even better than when we were there.” And that is the ultimate success of all….we’re taking care of sick people either through our own direct clinical operations or teaching the next generation of health care providers. (Participant 1-campus/university level)

### Individual Value

Organizational leaders viewed the benefits to the individual recipients as enhancing their development, career, and stature. Faculty develop skills and expertise as educators and leaders. Such initiatives serve as a launch pad for involvement in medical education, especially junior faculty who, if supported early on, can be engaged to have long successful careers, including accelerated promotion and leadership roles. Leaders underscored the affirmational impact that educator investments have on individuals. The honor of receiving an endowed chair or education grant is meaningful and gives educators credibility to do work about which they are passionate. Entrusting faculty with these funds gives them the flexibility to experiment with ideas, gain confidence and a desire to succeed, and do more beyond the duration of the funding. Leaders repeatedly highlighted that entrusting and investing in people also reaps rewards in domains beyond the individual to the operational, strategic or political, and social or societal levels of impact.

### Financial Value

Leaders acknowledged that EIPs have both practical and philosophical benefits. On a practical level, funding gives faculty permission and accountability to accomplish new educational projects. It protects faculty time and provides tangible resources for projects that cross departments or programs. Leaders saw these programs as a means to encourage additional resources from departments, clinical sites or the institution through matching contributions. Leaders also highlighted a deeper philosophical benefit of educational funding, emphasizing the importance of investing in education even though the result is not revenue generation.

### Operational Value

Leaders described 3 areas of operational impact that were of value: educational programs, efficiency, and recruitment and retention. Leaders viewed EIPs as important contributors to shaping, transforming, and improving teaching methods. They feed directly into activities that engage learners in cutting-edge science and prepare them for successful careers. Innovative programs and opportunities to participate in education projects attract the best learners and offer exposure for disciplines traditionally lacking visibility within medical education. Some leaders described the benefit of a collaboration of interprofessional educators who bring a broader spectrum of disciplines and social issues into the medical curriculum. Leaders noted that these programs contribute to meeting accreditation standards. They create opportunities for changes in the way that education is delivered, which might not otherwise be achieved because of limited resources. Leaders also saw improved efficiency as an important value. This included having more cost-effective educational programs and educating learners to enhance efficiency and decrease waste in clinical systems. Leaders highlighted recruitment and retention of faculty and learners as a critical operational value. EIPs help attract and recruit the best faculty and encourage them to stay at the institution. EIPs draw high-quality learners to the institution, often enabling future faculty appointments.

### Social and Societal Value

Leaders described societal value beyond the organization and social value to the organization’s internal community, including faculty, learners, and patients. An important value was enabling work within education that is generalizable and disseminated beyond the institution through presentations and publications. They wanted faculty to be leaders or experts in education scholarship that benefits patients and society and enhances education programs beyond the institution. Leaders highlighted the importance of investments that build connections to the community outside the institution and benefit the greater societal good. They gave examples of educators and programs with impact ranging from local to national and global levels. Leaders recognized the importance of EIPs that address pressing societal issues, reduce health care disparities, and improve access to care for diverse patient populations. They highlighted the institution’s social responsibility to train health care providers to deliver the best care.

Leaders recognized that networks and collaborations created through these initiatives benefit educators who learn from each other across departments and schools. These professional relationships support innovation and the confluence of ideas through a community of educators and scholars. They provide a forum to mentor junior educators, attract learners, and model best practices. These connections are important ways for individuals to experience a sense of belonging as part of a community of educators within the institution. Leaders described a desire for EIPs to support the development of educational programs that improve patient care. They felt that supporting educators lead to increased engagement and enthusiasm, which transferred to their patient care.

### Strategic and Political Value

Leaders deemed investing in education as strategically and politically important, which benefits organizational culture, innovation, and success. Investing in education has a symbolic impact for individuals, the organization, and beyond. Leaders expressed how committing to these programs recognizes the importance of education and education scholarship. Rewarding educators’ excellence and achievements ensure their visibility within the institution. It sends a message that educators’ work is crucial and equal to other missions. Awards bring prestige to recipients’ departments and leaders, raise departments’ education visibility, and contribute to the institution’s reputation. Leaders described the power to transform culture from one that undervalues educational contributions to one where education faculty feel welcomed, engaged, and empowered.

Innovation is an important strategic value factor expressed by leaders to keep the institution at the forefront of excellence. They felt these resources allowed for thinking creatively, taking risks, experimenting, and generating new knowledge. They prized having a protected space for educators to think, read, and dive deeper. Leaders expressed that an academic medical institution is great because of its educational programs, not the reverse. Education innovation, creativity, projects, and collaborations enable the success of both individuals and the entire institution. It is important that the work be aligned with the institution’s wider mission.

### Most Important Value Factors

Many leaders identified value factors that relate to enhancing both individual educator careers and professional development and the education programs as most important. One participant stated that “the human factor is the most important factor…investing in your people is the right thing to do, and it has wonderful outcomes.”

## Discussion

Using the VMM framework, we identified factors that academic institutional leaders value for investing in educators and education innovation. Leaders saw value in EIPs and emphasized benefits beyond financial ROI across all domains and illustrated that small financial resources can have substantial reach (Figure). Our findings are congruent with literature about health professions education intramural seed grant programs^3,14,15,16,17,18,19,20,21^ and endowed chair programs and are unique in that they represent the leader perspective.^4,22,23^ The positive impact described in the literature includes career growth, personal, and professional development, recruitment and retention (particularly with regards to increasing the diversity of faculty), education programs, collaboration, education scholarship and dissemination, innovation, and culture.^3,4,14,15,16,17,18,19,20,21,22,23^

**Figure.**
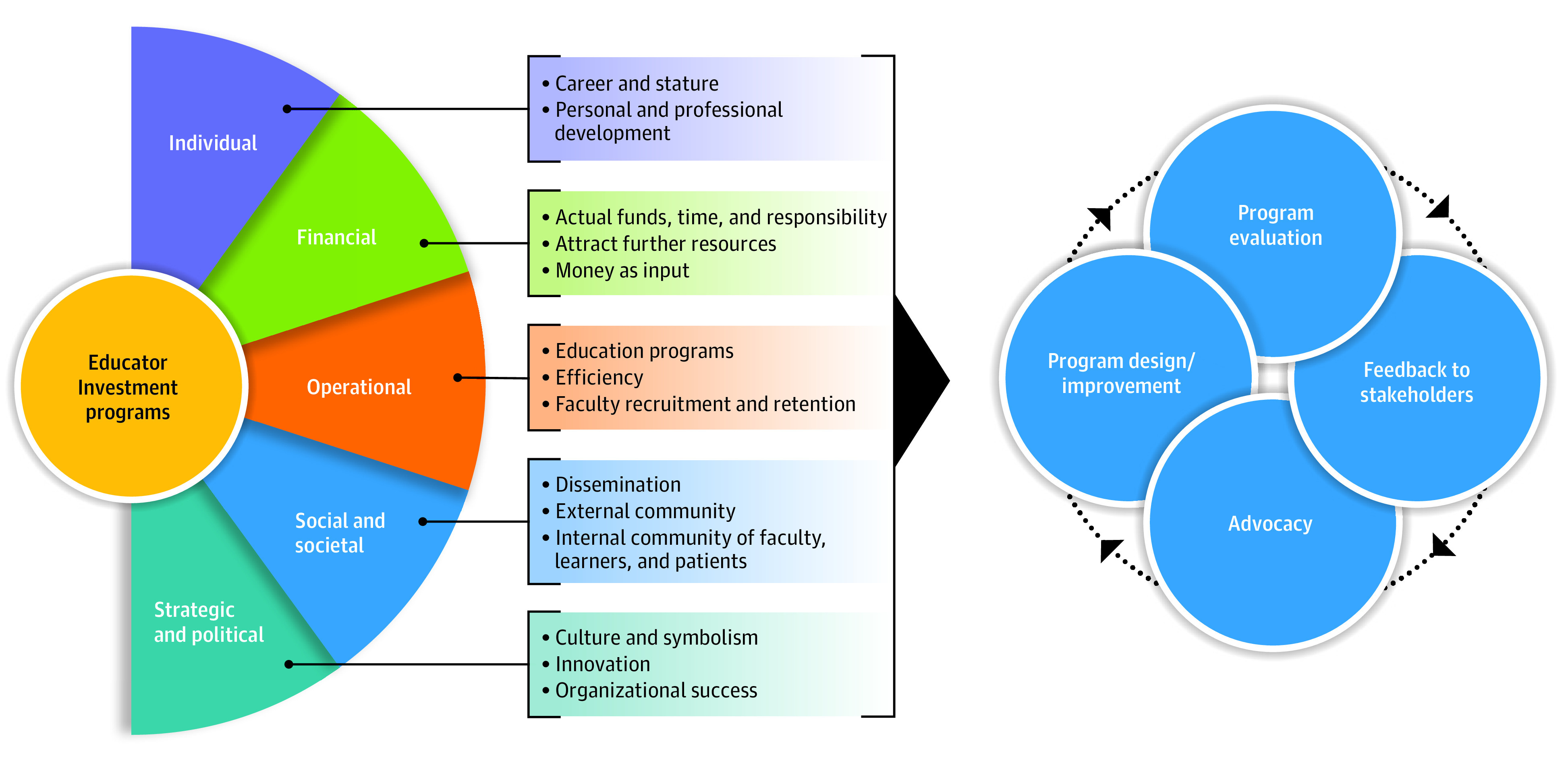
Value Domains and Identified Value Factors

Our leaders strongly value the impact of EIPs on individual recipients’ career growth and professional development. They highlighted the operational value of recruitment and retention. This aligns with Bolman and Deal’s human resource organizational principle of “investing the time and resources necessary to develop a cadre of committed, talented employees.”^24^ Designing EIPs to support educator careers can have considerable impact on job satisfaction and engagement, which is linked to well-being.^25,26,27,28,29,30^ Studies show that physicians who experience burnout are more than twice as likely to leave,^30^ and estimate the cost for replacing a physician at 2 to 3 times the physician’s annual salary.^28^ It is less expensive to invest in the development and engagement of health care providers than to replace them.^28,30,31^ Communicating the positive impact of EIPs on faculty careers, recruitment, and retention may encourage leaders to provide new or ongoing resources for EIPs.

Investing tangible resources in the career and development of individuals can have further impact at the institutional level on the success of the organization.^3,4^ Many of our leaders described as a most important value factor the positive effect of EIPs on educational program improvement and innovation. This operational outcome lends itself well to EIP program evaluation and documenting impact. Leaders appreciated the symbolic influence of EIPs on the organizational culture. They described the importance of making a statement about the value of education for the community of learners, faculty, and patients. This aligns with Bolman and Deal symbolic frame, which argues that symbols reflect and influence institutional culture and values.^24^

A pure economic model is too narrow a tool to measure valued outcomes from the academic education mission.^7^ A broader framework forms the basis for more effective dialogue between institutional leaders and educators. Applying VMM domains to an academic institution allowed us to better understand institutional leaders’ value parameters beyond traditional financial return on investment. Jabbar et al^7^ proposes economic imperialism as a concept that underlies the “power [of economics] over so many facets of social life and policy—including education” and drives the metrics we use to guide resource allocation. Understanding and acknowledging this hegemony allows policymakers to “approach decision-making through alternate frameworks and models.”^7^ The perspective in Jabbar et al^7^ underscores the importance of a study such as this to move beyond a traditional ROI model to a more holistic one within the current funding models for academic medical centers, which is driven by clinical revenue. Defining these metrics in a framework beyond financial can drive resource deployment and guide advocacy with language that speaks to the culture and a wider context of social factors.

Our study’s strengths include a broad representation of leaders’ opinions at various echelons of our institution. Many value factors elucidated by our leaders are generalizable across institutions, particularly the benefit to individual careers, recruitment and retention, educational programs, and scholarship. The VMM framework can be adapted and used in other institutions to explore context-specific value factors from local stakeholders. For example, innovation is a core mission at this institution, and our leaders connected it to the organization’s success. Other schools may articulate core missions of optimal patient engagement or advancing public health. Aligning EIPs with each school’s unique mission can engage leadership support for resources to initiate and sustain such programs. Value factors can differ between leaders at the same institution. Defining these value factors can inform how we design and evaluate EIPs, provide feedback to leaders about the value and impact of these programs and advocate for new or sustained funding (Figure).

### Limitations

This study had limitations. Our findings are limited by the dynamic shifts due to the coronavirus pandemic and social justice movements that occurred since our interviews. It would be of interest to know if leaders would include additional pertinent values if interviewed today. As researchers, we made every effort to report the true voices of the participants, acknowledging that many on the research team have been past grant or chair recipients. In reporting findings co-constructed through the interaction between researchers and participants, these perspectives also enhanced our data interpretation. Our findings represent a single institution supportive of the education mission; while many themes may be broadly applicable, the definition of value could vary based on local contexts. Future studies might explore leaders’ investment decisions in the context of competing priorities.

## Conclusions

These findings suggest that health sciences and health system leaders found value in funding educator investment programs in multiple domains (individual, financial, operational, social or societal, and strategic or political) beyond direct financial return on investment. They perceived that tangible resources to support the growth and professional development of individual educators generate potent benefits to the organization and community. These values can inform program design and evaluation, effective feedback to leaders, and advocacy for future investments in the context of competing priorities in academic medicine. This approach can be used by other institutions to identify context and leader-specific value factors from their leaders.

## Notes

Supplement 1. **eAppendix.** Interview Guide

Supplement 2.
Data Sharing Statement
