# Supplementary material for: Identifying Value Factors in Institutional Leaders’ Perspectives on Investing in Health Professions Educators
Source: JAMA Netw Open. 2023 Feb 16;6(2):e2256193. doi: 10.1001/jamanetworkopen.2022.56193 (PMC9936339; doi:10.1001/jamanetworkopen.2022.56193)
Supplement: Supplement 1. — eAppendix. Interview Guide [file jamanetwopen-e2256193-s001.pdf]

Supplemental Online Content

Poncelet A, Collins S, Fiore D, et al. Identifying value factors in institutional leaders' perspectives on investing in health professions educators. *JAMA Netw Open*. 2023;6(2):e2256193. doi:10.1001/jamanetworkopen.2022.56193

eAppendix. Interview Guide

This supplemental material has been provided by the authors to give readers additional information about their work.

© 2023 Poncelet A et al. *JAMA Network Open.*

eAppendix. Interview Guide

<u>Interview guide</u>**: Institutional Leaders' Perspectives on Investing in Health Professions Educators: Identifying Value Factors**

Introduction:

The Academy of Medical Educators (AME) stewards a number of competitive awards for faculty members to support education innovations and educator careers for 1-5 years at approximately $15,000-$30,000 per year through the Innovations Funding and Endowed Chair programs. Our primary aim is to explore our institutional leaders' perspectives on what factors are important when assessing the value and impact of these kinds of programs. This builds on several previous studies to assess the impact of these programs on the individual recipients

Here is a **list** of educators who have been selected for these programs from your **unit/department/school/health system** and the funding that they have received from AME. We are asking you to speak from your **XXX role** perspective. (comment: for leaders with dual roles, will ask about their perspective from each role)

Questions:

1. Please state your name and current/former role(s). How long have you been in the current role?

2. From your leadership perspective, what experiences have you had with these programs that were valuable?

    a. Can you define the factors (characteristics) of those experiences that made it valuable (impactful)?

       Probe: can you describe factors beyond individual impact?

    b. What value for your division/site/department/school/UC Health/UCSF and for the overall institution (depending upon the leader's role) is important for you, and if so, what factors would demonstrate that?

       Probe: From your leadership perspective, are there operational measures of value regarding impact of these programs that are important for you?
       Definition (if asked or as needed to focus/redirect responses): Operational measures are defined as improvements to the operations of the unit/department/school/health system, or lowering barriers to future initiatives. Simply put, this refers to getting the work of the unit/department/school/health system done.
       Examples: Increased efficiency, improved education systems, creating and/or running existing or new programs, quality improvement.

       Probe: From your leadership perspective, are there financial measures of value regarding impact of these programs that are important for you?

© 2023 Poncelet A et al. *JAMA Network Open.*

Definition: Financial measures include revenue, costs.
Examples: salary support, funds for professional development.

Probe: From your leadership perspective, are there strategic/political measures of value regarding impact of these programs that are important for you?
Definition: Strategic/Political measures are defined as efforts to advance the division/site/department/school/UC Health/UCSF's mission, strategic goals, priorities and mandates.
Examples: increase visibility of the education mission, enhanced support for educational activities, increase number of trained specialists in a discipline, be the highest ranked department/institution nationally.

Probe: From your leadership perspective, are there social or societal measures of value regarding impact of these
programs that are important for you?
Definition: Social or societal measures are defined as benefits to a larger group of people, such as a unit, department, school, or health system. Or benefits to a greater community, such as a population of patients, students, or faculty or to society in general.
Examples: Benefits to trainees, such as students or residents, increased recognition of educators, increasing engagement of faculty/staff, enhanced collaboration of educators across the institution.

c. From your leadership perspective, is there value for the individual recipient that is important for you, and if so, what factors would demonstrate that?
Examples: identity as an educator, improved skills, professional experience and growth, education scholarship and dissemination, enhanced reputation within and/or outside of the division/department/school/etc.

3. Of all of the factors you have described, which are the most important and why (could prompt with measures that the leader raised during the interview if needed)?

4. Is there anything else you would like to share?

5. What questions do you have?

© 2023 Poncelet A et al. *JAMA Network Open.*
