# Supplementary material for: Identifying Value Factors in Institutional Leaders’ Perspectives on Investing in Health Professions Educators
Source: JAMA Netw Open. 2023 Feb 16;6(2):e2256193. doi: 10.1001/jamanetworkopen.2022.56193 (PMC9936339; doi:10.1001/jamanetworkopen.2022.56193)
Supplement: Supplement 2. — Data Sharing Statement [file jamanetwopen-e2256193-s002.pdf]

Data Sharing Statement

Poncelet. Identifying Value Factors in Institutional Leaders' Perspectives on Investing in Health Professions Educators. *JAMA Netw Open*. Published February 16, 2023.
doi:10.1001/jamanetworkopen.2022.56193

Data
Data available: No

Additional Information
Explanation for why data not available: This is a qualitative study. Sharing the primary interview data would de identify the study participants. They provided consent with the expectation that they would remain anonymous
